# Masks wearing off: Changing effects of face masks on trustworthiness over time

**DOI:** 10.1177/03010066241237430

**Published:** 2024-03-07

**Authors:** Julian A. Oldmeadow, Taylor Gogan

**Affiliations:** 3783 Swinburne University of Technology, Australia

**Keywords:** trustworthiness, masks, COVID-19, person perception

## Abstract

During the global COVID-19 pandemic, the wearing of face masks became a common practice, raising questions about how masks affect perceptions of and behaviour towards others. Numerous studies have explored the impact of face masks on perceptions of trustworthiness, but results have been mixed and it remains unclear whether masks influence perceptions via their social meaning or their effects on facial appearance. In this study, Australian participants (*N *= 363) rated a series of faces which were either masked, unmasked, or occluded by a non-mask object (computer) in terms of perceived trustworthiness in 2020, 2022, or 2023. The apparent trustworthiness of unmasked faces remained stable across years, but masked faces were rated significantly more trustworthy in 2020 compared to 2022 and 2023. Furthermore, ratings of masked faces, but not unmasked faces, were correlated with participants’ attitudes towards wearing masks. Faces occluded by a non-mask object were perceived to be less trustworthy than masked faces. Together, results strongly suggest the increase in perceived trustworthiness of masked faces reported in numerous studies conducted during COVID-19 were driven by positive social meanings around mask wearing rather than by their effect on facial appearance.

The spread of COVID-19 across the world, beginning in early 2020, led to some significant changes in social behaviour. People maintained greater physical distance from others, stayed and worked from home more often, changed hygiene practices, and wore face masks when in public. Of these behavioural changes, the wearing of face masks has attracted considerable research attention, since relatively little was known about how face masks affect social perception and cognition. Since 2020, there has been a flurry of research activity assessing the impact of masks on social psychological processes, including recognition of identity and emotional expression (Carbon, 2020; Grundmann et al., 2021; Gülbetekin et al., 2023; Lau & Huckauf, 2021; Marini et al., 2021), trait impressions (Biermann et al., 2021; Gabrieli & Esposito, 2021; Oldmeadow & Koch, 2021; Olivera-La Rosa et al., 2020) and social interactions (Crandall et al., 2022). There are now review papers (Pavlova & Sokolov, 2022; Wang et al., 2023) and a research topic in Frontiers that includes a collection of 19 articles centred on the impact of face coverings on social cognition (Pavlova et al., 2023).

One foci for research into the social and perceptual effects of face masks has been their impact on the perceived trustworthiness of the wearer. Concerns that face masks may have a negative impact on social interaction and cohesion were aired in the media as well as in the scientific literature (Howard, 2020). These concerns were based on the belief that face masks may hinder communication and the ability to read emotional expressions, and that they may carry negative associations (e.g., with criminality or illness). There was very little research on this topic prior to COVID-19, but since the start of the pandemic there have been more than a dozen papers published that have directly assessed the effects of mask wearing on perceived trustworthiness, approachability or threat.

The results of these studies, though, have been mixed. Of 15 papers published between 2020 and 2023, six found masked faces were rated higher in trustworthiness compared to unmasked faces (Di Crosta et al., 2023; Guo et al., 2022; Kawakami et al., 2023; Lau, 2021; Oldmeadow & Koch, 2021; Olivera-La Rosa et al., 2020), four found masked faces were less trustworthy (Biermann et al., 2021; Bylianto & Chan, 2022; Gabrieli & Esposito, 2021; Takehara et al., 2023), and five found no differences or were inconclusive (Bennetts et al., 2022; C. Twele et al., 2022; Cannito et al., 2022; Grundmann et al., 2021; Oliveira & Garcia-Marques, 2022). The variability in results may stem from differences in methodology, as well as contextual factors that influence the meanings given to mask wearing (Wang et al., 2023). Specifically, the methodologies of these studies differ in terms of their operationalisation of trust (e.g., trustworthiness ratings, following advice of an individual), type of stimuli (e.g., static vs. dynamic faces, digitally overlaid masks vs. actual masks) and facial expressions of the stimuli. Additionally, studies have found very different responses to mask wearers in the US depending on the perceiver's political party affiliation (Powdthavee et al., 2021). Therefore, the effect of masks on trustworthiness judgements may vary depending on the perceiver's attitudes or meanings given to masks, over and above their perceptual effects. Given the different experimental paradigms, times and locations of the various studies, no clear picture has emerged regarding the influence of face masks on trustworthiness judgments.

In addition to mixed findings, there is no consensus over the mechanisms through which masks might impact trustworthiness judgments. Given that perceptions of trustworthiness are largely signalled from cues to the mouth (i.e., smiling), some have suggested the impact of masks may stem from the obstruction of facial cues and subsequent uncertainty or ambiguity in facial expressions. For example, several researchers have speculated the uncertainty induced by face masks may reduce trustworthiness (Gabrieli & Esposito, 2021; Grundmann et al., 2021). On the other hand, Guo et al. (2022) speculated that the same ambiguity ‘might induce a positivity bias in face evaluation process… and subsequently increase the perceived trustworthiness and approachableness’ (p. 46). In a somewhat more developed theory, Kramer and Jones (2022) argue that in the absence of a full face, perceivers ‘complete’ the face using facial stereotypes, which may lead to higher or lower perceived trustworthiness depending on the trustworthiness of the unobstructed face and the stereotype applied. Consistent with this, several studies show the effect of masks varies depending on the facial expression or trustworthiness of the face. Oldmeadow and Koch (2021) and Marini et al. (2021) found low trustworthy faces received a significant boost in trustworthiness when masked, whereas high trustworthy faces did not. Bylianto and Chan (2022) found faces with a happy expression were rated lower in trustworthiness when masked than unmasked. Thus, there is some evidence that masks affect perceived trustworthiness by obstructing facial cues normally used to make such judgments, but the precise nature of this effect is unclear.

If masks affect trustworthiness by obstructing or altering facial cues, one would expect similar effects from other forms of face occlusion. One study that blocked out specific regions of the face using a black rectangle found that trustworthiness judgments were higher when the nose or mouth regions were obscured (Santos & Young, 2011). Grahlow et al. (2022) examined perceptions of threat (similar to trustworthiness) across different facial expressions and different face occlusion conditions (a mask; showing only the top half of the face; obstructing the lower part of the face with a circle). They found very similar effects whether only the top half of the face was shown or the face was covered with a mask, but faces obscured by a circle were generally less threatening than those covered by a mask. In another study, Marini et al. (2021) compared the effects of opaque masks with that of transparent masks (which preserve at least some facial cues) and found both types of masks increased the perceived trustworthiness of low trustworthy faces, with opaque masks having a larger effect than transparent masks. These studies suggest the effects of masks can be partially, but not entirely, attributed to the occlusion of facial cues.

Other explanations for the effects of masks point to the social meaning of mask wearing and/or perceivers’ attitudes, particularly during the pandemic. Using interview data from several European countries, Schönweitz et al. (2022) found the meaning of mask wearing changed drastically around the onset of COVID-19, when it came to be associated with, among other things, moral duty. Olivera-La Rosa et al. (2020) speculated that the positive effect of masks on trustworthiness judgments found in their study might be due to an “internalized social norm of wearing a mask” (p. 5). Several studies provide evidence that perceivers’ attitudes influence their perceptions of mask wearers. Leder et al. (2022) found participants with more positive attitudes towards COVID-19 preventative measures gave higher ratings of pro-sociality to masked faces compared to unmasked faces. Di Crosta et al. (2023) found trustworthiness ratings of unmasked faces during the pandemic, which were lower than masked faces, were explained in part by raters’ fear of COVID. In a study conducted using a Japanese sample, Miyazaki and Kawahara (2016) found masked faces were rated lower in attractiveness, and this deficit was related to the belief that masked faces were less healthy. It is important to note that this study was conducted prior to COVID-19 and assessed attractiveness rather than trustworthiness, but it serves to demonstrate that perceivers’ assessment of the meaning of mask wearing can influence trait judgments of masked faces. It is likely that the effects of masks, particularly the positive effects on trustworthiness reported in a number of studies conducted during COVID-19, were at least in part due to the meanings given to the behaviour of mask wearing.

To summarise, despite considerable research examining the effects of masks on trustworthiness judgments, results have been mixed and there is no consensus on the mechanisms through which masks impact these perceptions. One approach that may help shed light on the issue is to examine how mask effects have changed over time while holding the methodological approach (e.g., operationalisation of trust, stimuli) constant. If effects are due to social norms around mask wearing, different effects may be observed over time as norms change. On the other hand, the perceptual effects due to obscuring facial cues would presumably not change over time.

A few studies have assessed changes in mask effects over time during the COVID-19 pandemic. Using UK-based samples, Bennetts et al. (2022) collected ratings of masked and unmasked faces across three time points between June 2020 and July 2021. They found no difference in trustworthiness between masked and unmasked faces and there was no effect of time. Takehara et al. (2023) examined trustworthiness ratings of masked and unmasked faces in mid-2020 and again in September 2022, using Japanese samples. Masked faces were rated lower in trustworthiness than unmasked faces, and there was no difference in this effect across the two time points. Di Crosta et al. (2023) compared trustworthiness of masked and unmasked faces during COVID and were able to compare scores for unmasked faces with ratings collected prior to COVID. Masked faces during COVID were rated more trustworthy than unmasked faces, which were rated lower in trustworthiness compared to pre-COVID, suggesting perhaps not wearing a mask during COVID was a sign of untrustworthiness. However, since there were no data on masked faces pre-COVID, it is difficult to draw firm conclusions about changes in the effects of masks from this study. In addition, these studies did not assess how attitudes changed over time, nor provide much contextual information about the status of mask wearing at different time points. Thus, the limited research examining mask effects over time does not enable clear conclusions to be drawn as to how masks affect trustworthiness and whether this has changed over time.

## The Current Research

The aim of the current study was to shed light on the effect of masks on trustworthiness and whether these effects are due to social norms around mask wearing during COVID, or to the obstruction of facial cues. We did this by comparing effects across time within the same social context, and comparing effects of masks to a non-mask occlusion. We compared trustworthiness ratings of faces across three time points from mid-2020 to mid-2023. The data for all three time points were based on ratings of the same set of faces and used very similar samples derived from the same population. Across all three time points we compared ratings of masked and unmasked faces. In the 2022 and 2023 time points (but not in 2020), we also included a condition in which faces were partially covered by a computer. This enabled a comparison of the effects of occlusion by a mask with those of occlusion by a non-mask object (devoid of social meaning), and thus informs whether mask effects are based on the occlusion of facial cues or on the meanings given to mask wearing. Also, in the latter two time points we measured participants’ frequency of mask wearing, attitudes towards wearing masks themselves, and attitudes towards others wearing and not wearing masks. These data inform whether ratings of masked faces were related to participants’ attitudes.

To give some context to the status of mask wearing in Australia throughout the time in which data was collected, before the COVID-19 pandemic face masks were uncommon in public and were mostly associated with medical practitioners (e.g., doctors, nurses, dentists). However, throughout large parts of 2020 and 2021, they became ubiquitous. In Victoria, masks became mandated in all public and outdoor spaces in July 2020 (Department of Health and Human Services, 2020). These restrictions were eased in February 2022, with masks required in certain settings such as hospitals, in taxis, and on public transport (Premier of Victoria, 2022). In September 2022 mask wearing became recommended but no longer mandated in any setting (Premier of Victoria, 2022b). Thus, during the first wave of data collection (21 August to 30 September, 2020) masks were mandated in all public and outdoor settings. During the second wave (17 April to 30 May, 2022) masks were only required in certain settings. During the third wave (17 May to 2 June, 2023), masks were not mandated in any setting.

## Hypotheses

The available evidence suggests that masks modulate trustworthiness judgements via the occlusion of facial cues (perceptual effects), and differences in social norms and meanings around mask wearing (contextual effects). The positive main effects of masks on trustworthiness reported in several studies conducted during COVID, in particular, may be best explained by social norms around mask wearing. If so, we would expect to see the positive effects of masks observed in our 2020 data (Oldmeadow & Koch, 2021) subsequently decline in 2022 and 2023 as the social norms around mask wearing dissipate.

Furthermore, this decline should only be seen for masked faces, not unmasked faces or faces covered with a non-mask object. Finally, we expected there would be an association between participants’ attitudes towards masks and their ratings of masked (but not unmasked) faces.

Based on the reasoning above, we put forward four hypotheses. First, there would be no differences in ratings of unmasked faces across all years (H1). Second, masked faces in 2022 and 2023 would be rated lower in trustworthiness than masked faces in 2020 (H2). Third, trustworthiness ratings of masked faces, but not of unmasked faces, would be correlated with participants’ attitudes towards mask wearing (limited to 2022 and 2023 data; H3). Finally, faces covered with a computer would be rated the same or lower in trustworthiness than faces covered with a mask (limited to 2022 and 2023 data; H4). This last hypothesis was based on the expectation that positive social meanings around mask wearing would persist to some extent in 2022 and, to a lesser degree, 2023, and so would contribute positively to perceptions of mask wearers compared to a non-mask object.

## Method

### Participants

In total, 363 participants (75.8% female, 22.6% male, 1.4% other) provided data at one of three time points between 2020 and 2023. Most participants were recruited from a pool of undergraduate psychology students at a large university in Melbourne, Australia. Others were recruited via social media advertisements. For ethical reasons we did not collect data on their location of residence, only their nationality. Three hundred and sixteen (87.1%) were Australian. Age ranged from 17 to 63 (*M *= 29.00, *SD *= 10.25). One hundred and eighty-two (50.1%) indicated their occupation as undergraduate students and 153 (42.1%) selected employed full- or part-time. The breakdown of sample size and demographic characteristics across the three time points is given in Table 1. Chi-square tests showed there were no differences across time points in nationality (*χ^2 ^*= 4.23, *df *= 4, *p *= .376) or occupation (*χ^2 ^*= 14.96, *df *= 14, *p *= .381). However, the gender ratio was not equivalent across time points (*χ^2 ^*= 12.29, *df *= 4, *p *= .015), with more females in 2020 compared to 2022 and 2023. In addition, the samples differed significantly in age, *F* (2, 352) = 7.11, *p *< .001, with the 2020 sample being older (*M *= 31.53, *SD *= 9.64) than the 2022 (*M *= 27.89, *SD *= 9.94) and 2023 (*M *= 27.08, *SD *= 10.63) samples.

**Table 1.** Sample size and demographic characteristics at each time point.

	2020 (21 Aug–30 Sept)	2022 (17 Apr–13 May)	2023 (17 May–2 June)
*n*	139	98	126
Age	31.53 (9.64)	27.89 (9.94)	27.08 (10.63)
Gender			
female	114 (82.0%)	77 (78.6%)	84 (66.7%)
male	24 (17.3%)	20 (20.4%)	38 (30.2%)
other	1 (0.7%)	1 (1.0%)	4 (3.2%)
Nationality			
Australian	122 (87.8%)	86 (87.8%)	108 (85.7%)
Other	15 (10.8%)	10 (10.2%)	14 (11.1%)
Occupation			
UG student	59 (42.4%)	50 (51%)	73 (57.9%)
Employed FT	41 (29.5%)	28 (28.6%)	31 (24.6%)
Employed PT	27 (19.4%)	13 (13.3%)	13 (10.3)

*Note*. The values for age denote mean scores with SD in parentheses.

### Materials and Procedure

#### Stimuli

**Figure 1.**
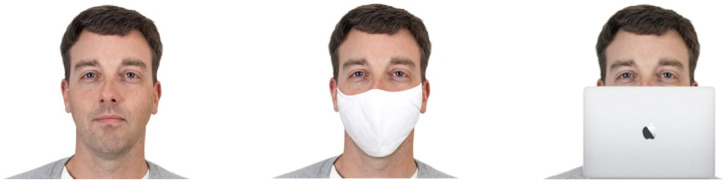
Example of a face images showing unmasked, masked and computer conditions.

The same face images were used at all three time points. A description of the stimuli was originally reported in Oldmeadow and Koch (2021) and is reproduced here. Forty faces were selected from the Chicago Face Database (Ma et al., 2015). Models were shown front-on with a neutral expression, against a plain background and wearing an identical plain t-shirt. Twenty Black faces and 20 White faces were selected, ensuring 50% male and female faces within each set. The norming data available for the face database was used to select the highest and lowest trustworthiness faces within each category. For example, the five most and five least trustworthy White male faces were selected for the White male subset, the five most and five least trustworthy White female faces were selected for the White female subset, and so on. Among the 40 chosen faces there were no significant differences in trustworthiness between male and female or Black and White faces, and no gender by race interaction (all *F*s < 1). Across the 40 faces, high trustworthy faces were significantly higher in trustworthiness (*M *= 4.15, *SD *= 0.24) than low trustworthy faces (*M *= 2.67, *SD *= 0.28), *t* (38) = 18.30, *p *< .001, Cohen's *d *= 5.79 (using norming data rated on a 7-point Likert scale). To create the masked images, a plain white mask was superimposed onto each face using Photoshop. For the computer condition, a laptop computer was superimposed onto each face image using Photoshop (see Figure 1).

#### Design

The study was a 2 (mask: no mask vs. plain mask) by 2 (face trustworthiness: high vs. low) by 3 (year: 2020 vs. 2022 vs. 2023) mixed design with repeated measures on the first two factors. The dependent variable was ratings of trustworthiness/approachability given to face images. Each of the 40 faces appeared once without a mask and once with a plain mask. In addition, the 2022 and 2023 data included a condition with the lower region of the face covered by a computer. As these conditions were not included in the 2020 data, they are analysed in a separate analysis below. Presentation order of all images was randomised across participants.

#### Procedure

The study was reviewed and approved by a university human ethics committee in accordance with the Helsinki declaration. Data were anonymous and approval was granted to provide open access via the Open Science Framework. The study was run entirely online. After providing informed consent participants read:

*On the following pages you will be shown a series of faces. Some of these will be wearing face masks and some will not. Your task is to rate each face in terms of how TRUSTWORTHY or APPROACHABLE the person appears. In this study we are interested in your first impressions, so please base your responses on your first impression or impulse about each face. You should aim to give a response to each face within one or two seconds*.

The terms trustworthy and approachable were combined because both terms have been used in previous studies to capture an underlying factor and are highly correlated (Sutherland et al., 2013). Each image was then shown one at a time in random order, above a 9-point response scale anchored with ‘not at all’ (1) and ‘very’ (9). After rating the faces, participants were asked to respond to basic demographic questions. In the 2022 and 2023 samples (but not in 2020), participants also answered several questions assessing their frequency of and attitudes towards wearing masks. One question asked how frequently they wear face masks in public (1 = never, 5 = always). Another question asked about their attitude towards wearing face masks (1 = very negative, 9 = very positive). Four further questions asked about their attitude towards others wearing or not wearing masks, for example, ‘If someone is wearing a mask in public, I think they are doing the right thing’, ‘If someone is not wearing a mask in public, I try to keep my distance from them’ (1 = strongly disagree, 5 = strongly agree). The four items measuring attitudes towards others wearing masks formed a reliable scale (Cronbach's *α *= .85), with higher scores indicating more positive attitudes towards others wearing masks. Finally, participants were presented with a study explanation and thanked for their time. All participants received course credit for their participation.

## Results

### Effect of Mask and Year on Perceived Trustworthiness

Ratings of perceived trustworthiness were analysed using a 2 (mask) by 2 (face trustworthiness) by 3 (year) mixed model ANOVA. Since gender differed across years, gender was initially included as an additional between-subjects factor to assess any gender effects (only male and female genders were included since there were insufficient numbers of ‘other’ genders to include in the analysis). The only significant effect involving gender was a gender by face trustworthiness interaction, *F* (1, 351) = 12.86, *p *< .001. Females differentiated more between high and low trustworthiness faces than males did. Since there were no interactions involving gender and mask or year, it was concluded the different gender ratios across years did not significantly impact the results. Therefore, gender was not included in the final analysis. A second preliminary analysis included age as a covariate, since age differed significantly across samples. There were no significant effects involving age, so it was concluded age differences did not impact on the results and was dropped from the final analysis.

In the final analysis there was a significant main effect of face trustworthiness, *F* (1, 360) = 1116.44, *p *< .001, η^2 ^= .76, with high trustworthy faces rated more trustworthy (*M *= 5.99, *Se *= .05) than low trustworthy faces (*M *= 4.40, *Se *= .06). There were also significant two-way interactions between mask and face trustworthiness, *F* (1, 360) = 90.09, *p *< .001, *η^2 ^*= .20, mask and year, *F* (2, 360) = 11.04, *p *< .001, *η^2 ^*= .06, and face trustworthiness and year, *F* (2, 360) = 8.45, *p* < .001, *η^2 ^*= .05. No other main effects or interactions were significant.

Regarding the interaction between mask and face trustworthiness, low trustworthiness faces were rated higher in trust when masked (*M *= 4.59, *SD *= 1.27) than unmasked (*M *= 4.21, *SD *= 1.34), *F* (1, 360) = 36.02, *p *< .001, whereas high trustworthiness faces were rated lower in trust when masked (*M *= 5.71, *SD *= 1.24) than unmasked (*M *= 6.12, *SD *= 1.07), *F* (1, 360) = 16.65, *p *< .001. The interaction between face trustworthiness and year was driven by higher ratings of high trustworthy faces in 2020 (*M *= 6.22) compared to 2022 (*M *= 5.83, *p* = .004) and 2023 (*M *= 5.93, *p *= .023).

**Figure 2.**
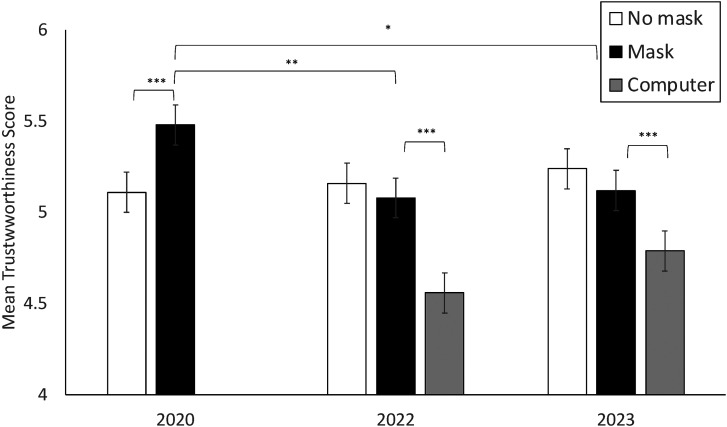
Mean trustworthiness across condition and year.

The interaction between year and mask is shown in Figure 2. Simple main effects analyses indicated no differences between years in ratings of unmasked faces, supporting hypothesis 1. However, ratings of masked faces were higher in 2020 (*M *= 5.48, *Se *= .097) compared to 2022 (*M *= 5.08, *Se *= .12, *p *= .008) and 2023 (*M *= 5.12, *Se *= .10, *p *= .012), supporting hypothesis 2. Furthermore, the difference between masked and unmasked faces was significant in 2020 only, *F* (1, 360) = 22.26, *p *< .001, *η^2^*= .06.

* p < .05; ** *p* < .01; *** *p* < .001.

### Effects of Attitudes on Perceived Trustworthiness

To assess whether participants’ behaviour or attitudes towards wearing masks impacted their trustworthiness judgements for masked faces, correlations were computed with ratings of masked and unmasked faces in 2022 and 2023 (see Table 2). Attitudes towards others wearing face masks were lower in 2023 (*M *= 2.49, *SD *= .82) than in 2022 (*M *= 3.20, *SD *= 1.05), *t*(218) = 5.63, *p *< .001, indicating participants were less concerned about whether others wore face masks or not in 2023 compared to 2022. Participants’ attitudes towards wearing masks themselves were also less positive in 2023 (*M *= 5.58, *SD *= 2.11) compared to 2022 (*M *= 6.24, *SD *= 2.23), *t*(218) = 2.27, *p *= .024. Participants reported wearing face masks themselves less frequently in 2023 (*M *= 1.47, *SD *= 0.56) than in 2022 (*M *= 2.50, *SD *= 1.02), corresponding to a change from between ‘sometimes’ and ‘about half the time’, to between ‘never’ and ‘sometimes’.

**Table 2.** Correlations between behaviour, attitudes, and trustworthiness ratings.

	Self att.	Behaviour	Unmasked	Masked	Mask diff.
Other att.	.666**	.625**	.000	.130^	.140*
Self att.		.474**	.046	.256**	.231**
Behaviour			.026	.038	.016
Unmasked				.530**	−.404**
Masked					.562**

Self att. = attitude towards wearing a mask; Other att. = attitude towards others wearing masks; Behaviour = frequency of wearing a mask; Mask diff. = difference between ratings of masked and unmasked faces.

** *p* < .001, * *p* < .05, ^ *p* = .054.

As expected, there were no significant correlations between behaviour or attitudes and trustworthiness scores of unmasked faces in 2022 and 2023 (*r*s < .05, *p*s > .502). However, there were significant correlations between participants’ attitudes towards wearing masks themselves and ratings of masked faces (*r *= .26, *p *< .001). The more positive participants were about wearing masks themselves, the more trustworthy they perceived masked faces to be. There also was a marginally significant correlation between ratings of masked faces and attitudes towards others wearing or not wearing masks (*r *= .14, *p *= .054). Finally, we created a difference score by subtracting ratings of masked faces from unmasked faces. This score was correlated with participants’ attitudes towards wearing masks themselves (*r *= .23, *p *< .001) and towards others wearing masks (*r *= .14, *p *= .038). Hypothesis 3 was partially supported.

### Effects of Masks Compared to Non-Mask Object

To compare the effect of masks with that of a non-mask object (computer), a 2 (year: 2022 v. 2023) by 2 (trust: high v. low) by 2 (condition: mask v. computer) mixed ANOVA was conducted. Of relevance to hypothesis 4, there was a significant main effect of condition *F* (1, 218) = 104.71, *p *< .001, *η^2 ^*= .32, and a two-way interaction between condition and year *F* (1, 218) = 4.14, *p *= .043, *η^2 ^*= .02. Faces covered with a computer were rated lower in trustworthiness than faces covered with a mask in both 2022, *F* (1, 218) = 67.85, *p *< .001, *η^2 ^*= .24, and 2023, *F* (1, 218) = 37.72, *p *< .001, *η^2 ^*= .15, though slightly more so in 2022 than 2023. The interaction between condition and face trustworthiness was not significant, *F* (1, 218) = 2.41, *p *= .122, *η^2 ^*= .01.

## Discussion

In this study, we compared the perceived trustworthiness of masked and unmasked faces across three time points to assess whether the effects of masks changed since the beginning of the COVID-19 pandemic in 2020. At each time point the same stimuli and procedure was used, and samples with similar characteristics were drawn from the same participant pool. Therefore, the major difference between time points was contextual changes in the threat of COVID-19 and the prevalence of mask wearing. We reasoned that if the effects of masks on perceived trustworthiness are primarily due to perceptual processes (the obstruction of facial cues), these effects should remain stable over time and should be similar to the effect of occlusion by a non-mask object. However, if they are due primarily to social norms and meanings around mask wearing, these effects may change over time and differ from a non-mask object.

The results show a clear difference in the perceived trustworthiness of masked faces in 2020 compared to 2022 and 2023. While unmasked faces received similar ratings across years, masked faces were perceived to be more trustworthy in 2020 compared to 2022 and 2023, confirming hypotheses 1 and 2. They also were perceived to be more trustworthy than unmasked faces in 2020, but not in 2022 or 2023. These changes clearly suggest the increase in trustworthiness of masked relative to unmasked faces in 2020, which was also found in a number of other studies conducted in the early stages of the pandemic (Di Crosta et al., 2023; Guo et al., 2022; Kawakami et al., 2023; Lau, 2021; Oldmeadow & Koch, 2021; Olivera-La Rosa et al., 2020), were driven by social norms and meanings around mask wearing rather than by perceptual effects. Further evidence for this comes from the correlations found between attitudes towards mask wearing and the perceived trustworthiness of masked (but not unmasked) faces, in line with hypothesis 3. Participants’ attitudes towards their own mask wearing and towards others wearing masks was associated with their ratings of the trustworthiness of masked faces, indicating trustworthiness ratings were linked to the behaviour of wearing a mask. Finally, trustworthiness ratings of masked faces were higher than those of faces occluded by a non-mask object, supporting Hypothesis 4. This indicates that masks continued to carry some positive effect in 2022 and 2023 due to their meaning, or it may be that faces occluded by a computer somehow appeared less trustworthy (as if they were hiding). Overall, this study provides strong evidence that the attribution of greater trustworthiness to masked than unmasked faces in 2020, reported in this and other studies, was due primarily to the social meanings given to masks and mask wearing rather than their direct perceptual effects.

Masks do nevertheless appear to have a perceptual effect on perceived trustworthiness, evidenced by the interaction between mask wearing and facial trustworthiness. However, the net effect was not to increase or decrease trustworthiness overall. Rather, masks reduced the difference between high and low trustworthy faces, with low trustworthy faces increasing and high trustworthy faces decreasing in perceived trustworthiness. By obstructing cues normally used to judge trustworthiness and diverting attention to less diagnostic facial cues (Bylianto & Chan, 2022), high and low trustworthy faces appear more similar in trustworthiness. Even for masked faces, though, there was a significant difference between high and low trustworthiness faces, indicating that facial cues other than those covered by a mask are used to judge trustworthiness.

The results of this study suggest that whether face masks have an overall positive or negative effect on perceptions of wearers likely depends on the social meaning of mask wearing in a particular context and perceivers’ attitudes towards masks. The direct perceptual effect of occluding facial cues likely makes it more difficult to judge traits like trustworthiness, just as it makes emotion recognition harder (Carbon, 2020; Grahlow et al., 2022; Marini et al., 2021), but this does not necessarily lead to higher or lower perceived trustworthiness overall. Rather, as Kramer and Jones (2022) argue, this depends on the characteristics of the unmasked face. In our data, the higher ratings given to masked faces in 2020 were likely due to positive social norms around mask wearing being a ‘moral duty’ (Schönweitz et al., 2022), as masks were mandated and there was considerable anxiety around COVID-19. In 2022 and 2023 mask mandates were relaxed, the wearing of and attitudes towards masks declined, and positive perceptions of mask wearers was wearing off.

### Limitations

A limitation of this study is that we have no direct evidence that the higher ratings of trustworthiness given to masked faces in 2020 compared to 2022 and 2023 was due to positive social norms around mask wearing. It is possible that the higher ratings were due to idiosyncratic differences between the samples, minor methodological differences between the samples (the 2020 sample also rated faces covered with a branded mask, while those in 2022 and 2023 also rated faces covered with a computer), or to changes in government policies around mask wearing. However, these alternative explanations seem unlikely. Firstly, the samples were recruited through the same channels from the same population and were very similar in demographic characteristics. Secondly, it is unlikely that ratings of the same masked faces across years would have been significantly affected by the other conditions included in the studies (a branded mask versus a computer). Finally, the fact that ratings of masked faces correlated with participants’ attitudes towards mask wearing provides further evidence that ratings in 2020 were likely to have been influenced by social norms.

Another limitation is that we did not collect data on participants’ attitudes in 2020. If we had, we could potentially have conducted a mediation analysis to test whether differences in ratings of masked faces across the years were due to changes in participants’ attitudes. Without that data, unfortunately, such an analysis was not possible. Nevertheless, it is notable that both the prevalence of mask wearing and participants’ attitudes declined significantly between 2022 and 2023. It is therefore not unreasonable to suppose attitudes were significantly more positive in 2020.

Another limitation concerns the nature of the stimuli used. Although it was essential to control for sources of variation in the faces other than the presence or absence of masks (or non-mask objects), to isolate their effect on trustworthiness judgements, it might be hard to generalise. Specifically, the stimuli used in the current study were highly standardised and presented on a plain background. However, COVID mandates in Australia were often context dependent; for instance, there were often different rules and social norms for wearing masks on public transport compared to walking your dog in the park. The mandates for different contexts generally reflected the relative risk of COVID transmission—people are more at risks of getting COVID in enclosed spaces. Arguably, the relationship between face masks and perceived trustworthiness would be stronger in high-risk (e.g., in a hospital) than low risk contexts (e.g., in a park). However, it is worth noting that embedding the stimuli with contextual information would have likely strengthened our conclusions.

We found that faces occluded by a computer were rated lower in trustworthiness than masked faces. However, it is unclear whether this was due to positive meanings given to mask wearing, negative meanings given to the faces occluded by a computer, or to minor differences in visibility of facial cues between conditions. Grahlow et al. (2022) found faces were rated equally threatening whether covered with a mask or only the top half was shown. We chose to occlude faces with a computer in order to maximise realism, but in hindsight, showing only the top half of the face may have been a cleaner test of the effect of occluding facial cues.

Finally, because the non-mask occlusion condition was not available in the 2020 data, we could not confirm that the positive evaluation of masked faces in 2020 was exclusive to face masks. If faces covered by a computer were rated similarly trustworthy in 2020 as in 2022 and 2023, this would have provided stronger evidence that the effects of masks in 2020 were related to positive social norms around mask wearing.

### Conclusion

In this study, we found that the boost to perceived trustworthiness of masked faces in 2020 was substantially reduced in 2022 and 2023, to the point where there were no longer differences between masked and unmasked faces in perceived trustworthiness. We also found that ratings of masked, but not unmasked, faces were correlated with participants’ attitudes towards mask wearing, and that masked faces were rated higher in trustworthiness than faces obscured by a computer. Together, these results provide evidence that the boost to trustworthiness of masked faces recorded in 2020 and also reported in a number of other studies, was likely due to positive social meanings given to mask wearing rather than to direct perceptual effects. Our results suggest that as the threat of COVID reduced and masks became less normative once again, the positive benefits of wearing a mask on person perception wore off so that now, wearing a mask does not necessarily make a person appear more (or less) trustworthy.
